# Multiple Evanescent White Dot Syndrome Presenting in Possibly Contracted Hand-Foot-and-Mouth Disease: A Rare Presentation

**DOI:** 10.7759/cureus.4903

**Published:** 2019-06-14

**Authors:** Lakmal S Ekanayake, Vikrant Bhatnagar, Philip A Bucur, Amandeep Goyal

**Affiliations:** 1 Miscellaneous, Ohio University Heritage College of Osteopathic Medicine, Athens, USA; 2 Family Medicine, Ohio University Heritage College of Osteopathic Medicine, Athens, USA; 3 Internal Medicine, Marietta Memorial Hospital, Marietta, USA

**Keywords:** mewds, multiple evanescent white dot syndrome, hand-foot-and-mouth disease, white dot syndromes, coxsackievirus, autoimmune, ophthalmology

## Abstract

Multiple evanescent white dot syndrome (MEWDS), an inflammatory retinal condition seen predominantly in young adult patients, is characterized by unilateral vision loss with variable scotomas. The etiology of MEWDS is currently elusive and the formal mechanism is unknown. However, it must be differentiated from other white dot syndromes (WDS). Fundus fluorescein angiography in MEWDS exhibits a “wreath-like” arrangement of hyperfluorescent lesions predominantly located in the outer retina. Herein we present a case of a 32-year-old Southeast Asian female who presented to the emergency department with peripheral blindness and central scotomas. The patient's daughter was diagnosed with hand-foot-and-mouth disease seven days earlier, which often presents as an extremely debilitating condition. Below, we elaborate on the etiology, pathogenesis, and diagnostic methods to elucidate the multifactorial causes of MEWDS and aid clinicians in diagnosis and treatment. Although associated with certain viral illnesses, to the best of our knowledge, there have been no reported cases of MEWDS in conjunction with hand-foot-and-mouth disease.

## Introduction

Multiple evanescent white dot syndrome (MEWDS) is a rare unilateral disease consisting of small white dots that spontaneously resolve. The American Academy of Ophthalmology states that MEWDS presents as a flu-like illness prior to the onset of ocular symptoms in 50% of cases [1]. This syndrome disproportionately affects women in the 3rd and 4th decades of life [2]. Because the natural history of the disease is restricted in time, many cases of MEWDS resolve without pharmaceutical intervention [3].

White dot syndromes (WDS) are a family of inflammatory ocular disorders with an unclear etiology that affect various structures of the eye such as the external retina, retinal pigment epithelium, and choroid [2]. When considering MEWDS, a differential diagnosis should include other WDS such as acute posterior multifocal placoid pigment epitheliopathy, acute zonal occult outer retinopathy, and optic neuritis [4]. Hallmark features that help providers distinguish MEWDS from other WDS include (1) patient demographics (females of child-bearing age), (2) flu-like illness preceding ocular symptoms, and (3) decreased vision and photopsia scotomas [5].

The lesions present in MEWDS are variable in size and range from numerous dots to large spots. Spots are a collection of small lesions arranged in clusters. The inflammation caused by MEWDS leads to dilation of the retinal vasculature and diagnostic imaging such as fluorescein angiography depicts a hyperfluorescent pattern in the deep retinal capillaries [6,7].

Although it is a self-limiting condition typically treated with supportive care, cyclosporine has been beneficial in recurrent cases [4]. Long term visual acuity and vision are favorable with the patient returning to disease-free values. The period of total recovery is approximately two months [7].

Research into possible viral causes precipitating MEWDS has been conducted, potentiating autoimmune origins [8]. While there have been several cases reported in the literature about the development of MEWDS following a variety of vaccine administrations [8-11], there have been no reports highlighting the clinical manifestation of MEWDS from hand-foot-and-mouth disease transmission, as we present here.

## Case presentation

A 32-year-old Southeast Asian female presented to the emergency department (ED) complaining of peripheral blurred vision and black blind spots appearing centrally in her left eye that began seven days prior. The patient’s daughter was clinically diagnosed with hand-foot-and-mouth disease seven days prior to the patient’s symptom onset. The patient’s surgical history was significant for cesarean section. Medical history was not significant for any acute or chronic conditions. The patient was referred to an ophthalmology out-patient office.

**Figure 1:**
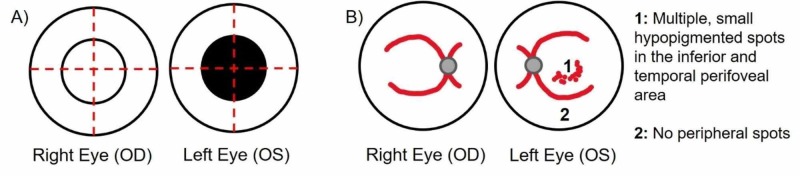
Patient's Visual Field Testing and Fundus Exam (A) The visual field which presents a central scotoma in the left eye (OS), and a normal field in the right eye (OD). (B) The fundus exam displays an enlarged optic disc in both eyes (OU), cup to disc ratio 0.5 (OU), and a normal macula (OD). Furthermore, multiple, small hypopigmented spots in the perifovea, most obvious inferior and temporal to the fovea, are seen (OS). Bl­ood vessels were normal (OU), periphery was normal (OD), and no lesions were present (OS). Outside fluorescein angiography showcases a “wreath-like” hyperfluorescent pattern in the macula (OS).

The patient was then seen in the ophthalmology office four days after her ED visit. On physical examination, visual acuity of the left eye was found to be 20/80 with an intraocular pressure (IOP) of 15 mmHg and 20/30 in the right eye with an IOP of 16 mmHg. She was given 1% tropicamide and 2.5% phenylephrine for dilation. Pupils were found to be equally round and reactive to light, have brisk reactivity, and without afferent pupillary defect. Extra-ocular movements were found to be full and orthophoric in both eyes. Importantly, a central scotoma was noted in the left eye but absent in the right eye (Figure 1A). Fundus exam of the left eye showed multiple small hypopigmented spots in the perifovea area, inferior and temporal to the fovea (Figure 1B).

**Figure 2:**
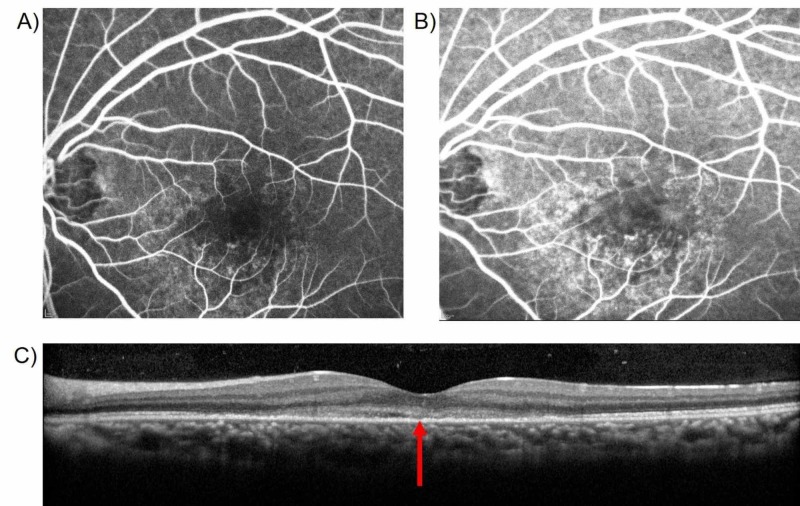
Fundus Fluorescein Angiography and Optical Coherence Tomography (A and B) Fundus fluorescein angiography illustrates the left eye with multiple, distinct hyperfluorescent spots located circumferentially around the fovea with a predominance in the inferior and temporal area of the perifovea. The foveola remains unaffected. No lesions were found in the right eye. Bl­ood vessels were normal in both eyes. (C) Optical coherence tomography of the left eye depicts the disruptions in the outer layers of the retina, indicated by the red arrow.

Fundus fluorescein angiography of the left eye exhibited multiple distinct hyperfluorescent spots located circumferentially around the fovea with a predominance in the inferior and temporal area of the perifovea (Figure 2). The foveola remained unaffected. No lesions were found in the right eye. Bl­ood vessels were normal in both eyes. Laboratory blood tests were not ordered in the emergency department and ophthalmology out-patient office.

**Figure 3:**
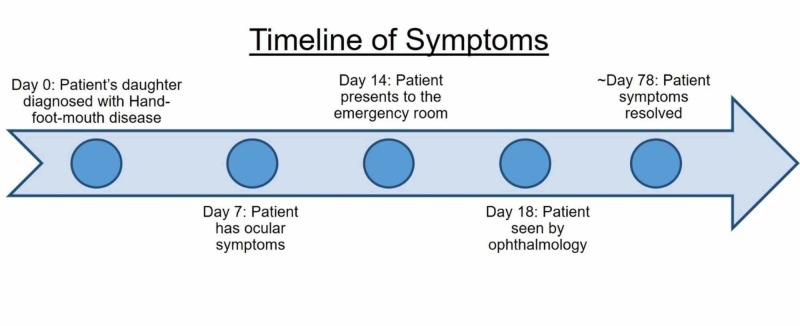
Patient's Course of Disease This timeline illustrates the patient’s symptoms from diagnosis of hand-foot-and-mouth disease in the patient’s daughter, to the onset of ocular symptoms, and to the resolution of the patient’s symptoms.

The patient was discharged and instructed to return in two months for dilation and Cirrus optical coherence tomography of the left eye. However, the patient had canceled her follow-up appointment but informed us that her symptoms had resolved one week prior to her scheduled visit. Figure 3 illustrates the patient’s timeline of symptoms, with resolution of ocular symptoms about 10 weeks after their onset.

## Discussion

Although lost to follow-up, the patient responded well to supportive care. The patient was asymptomatic without any changes to her vision within seven weeks of visiting the ophthalmology office. The etiology of MEWDS is an uncommon one and, for the purpose of this case presentation, we deduce an infectious source. The ability to deduce the life cycle of the viral insult is helpful but not always feasible for clinicians. Physicians must develop a differential that includes MEWDS in addition to more common eye conditions, such as multiple sclerosis, diabetic retinopathy, and neoplastic vascularization.

This case represents the importance of elucidating a thorough history for a susceptible population, such as women of childbearing age. Given that the patient has a small child who may have been exposed to various infectious agents in a variety of settings (such as daycare, interacting with other children, and a developing immune system), we should especially focus on the origin and transmission of the viral insult.

To note, hand-foot-and-mouth disease is caused by Coxsackievirus, a mildly contagious viral infection belonging to the viral family Picornaviridae [12]. There have been several cases that have linked other viruses including picornavirus vaccination to MEWDS. One such case describes MEWDS following the hepatitis A vaccine [9]. In that case, a 30-year-old male had received a hepatitis A vaccine booster and developed worsening vision in his left eye after 13 days. He was diagnosed with MEWDS after findings of unilateral vision loss, photopsias, an enlarged blind spot, and numerous yellow-white dots occurring on the outer retina, all appearing after hepatitis A vaccination [9]. That case further hints at the immune-mediated etiology of MEWDS. In our case presentation, we describe a patient presenting with MEWDS after possibly contracting the Coxsackievirus, adding to the literature another manifestation of the complex interplay between an environmental trigger and an autoimmune-mediated disorder.

## Conclusions

MEWDS is a significant diagnosis, which presents as an extremely debilitating condition for patients. In lieu of proper diagnostic tools, clinicians and scientists still face a tremendous obstacle in fully uncovering the etiology of this multifactorial syndrome. Clinicians must collect in-depth histories from patients to adequately diagnose and treat them. Importantly, the use of imaging modalities including fluorescein angiography, basic fundus exam and optical coherence tomography can support and confirm the diagnosis of MEWDS. Currently, treatment is supportive in addition to close monitoring for further exacerbations or new onset of visual symptoms.
